# Supplementary material for: Enhancing Endocannabinoid Signaling via β-Catenin in the Nucleus Accumbens Attenuates PTSD- and Depression-like Behavior of Male Rats
Source: Biomedicines. 2022 Jul 25;10(8):1789. doi: 10.3390/biomedicines10081789 (PMC9394396; doi:10.3390/biomedicines10081789)

Supplementary Materials

Figure S1. Antibody specificity

Demonstration of antibodies and their specificity: (a) Anti CB1r {predicted molecular weight (PMW): 53 kDa, abcam, UK; ab25932 [ERP23934-20]}; (b) anti β–Catenin (PMW: 94 kDa; abcam, UK; ab32572 [E247]); (c) anti mGluR5 (PMW: 132 kDa; abcam, UK; ab76316 [ERP2425Y]); (d) β-actin (PMW: 45 kDa; Cell Signaling, USA; #5125 [13E5]).

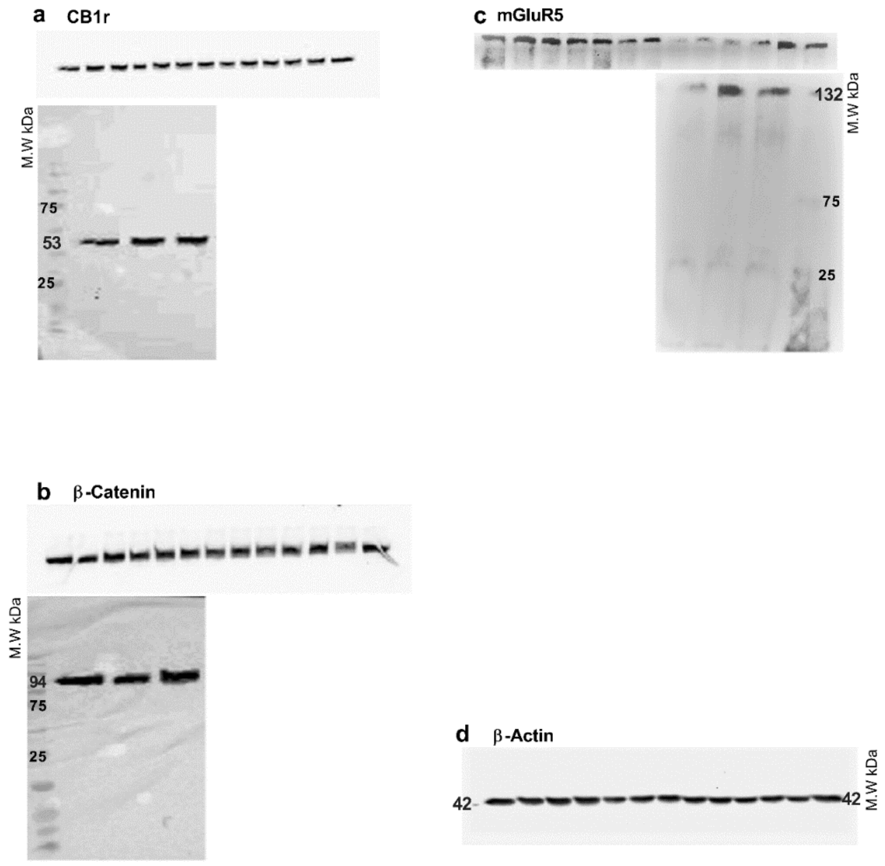

**SuperMarker2700 ; Cat# 9597580SM2700 ; Bio-Lab Israel

Figure S2. An illustration of a modified HSV amplicon plasmid

An illustration of a modified HSV amplicon plasmid with an added transcription cassette expressing GFP, producing a separate transcript [i.e., promoter and poly(A)] for GFP (provided by Nestler's lab). The target gene is still driven by the IE 4/5 promoter, while the GFP is driven by a CMV promoter. The two transcription cassettes are in a nose-to-tail orientation. Co-expression is generally above 90%. In the short-term experimental design, HSV has advantages over many other viral vectors, since the viral expression in vivo lasts only about 8 days (see, e.g., Neve et al., 2005).

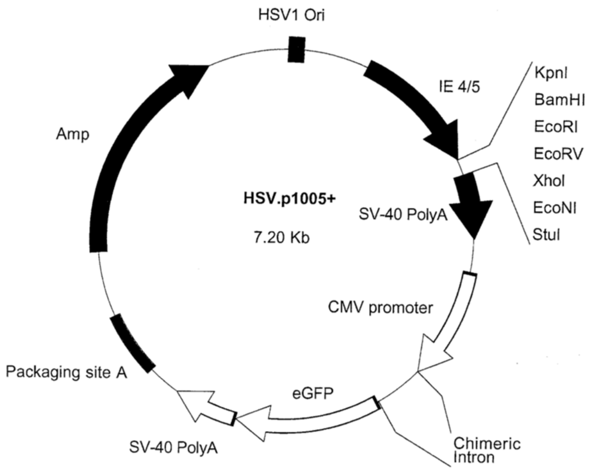

Figure S3. The effects of URB597 on exploration time during the social preference and the social recognition tests

A two-way ANOVA (shock×drug; 2×2) on total exploration time revealed a significant main effect of shock in social recognition ($F(1,36)= 4.606$, $p<0.05$) as well as a drug×shock interaction for social preference ($F(1,36)= 6.56$, $p<0.05$).

Post hoc analysis revealed that during the social preference test, the Shock/Veh group demonstrated decreased exploration time compared with the NoShock/Veh and Shock/URB597 groups (both $p<0.05$). During the social recognition test, the Shock groups showed a significant decrease in exploration time compared with the NoShock groups (all $p<0.05$). Hence, URB597 restored the shock- and reminders-induced decrease in total exploration time in the social preference task but not in the social recognition task. (*, $p<0.05$)

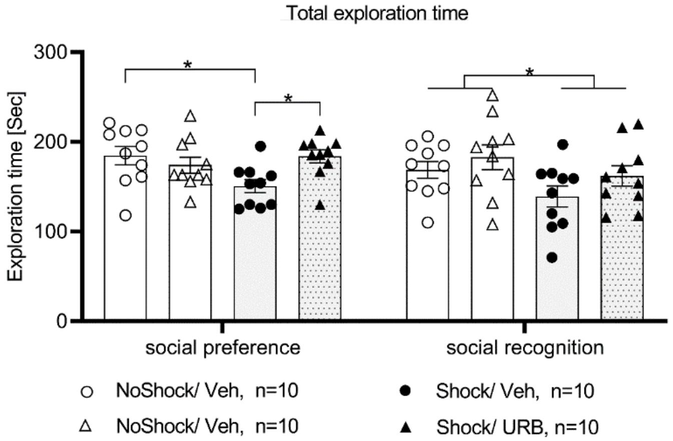

Table S1. Pearson bivariate correlation between the expression of β-catenin and behavior

Task	NAc	mPFC
ASR	**-.461****[1]	-.300
Social preference	.291	.117
Social recognition	**.367***	.255
T- maze acquisition	.291	.117
T- maze reversal	**.367***	.255
FST immobility	-.302	-.100
FST climbing	**.439****	**419****
FST swimming	-.084	-.021

[1] Significant correlations were found between NAc β-catenin levels and the following behaviors: ASR: (r = -0.461, p<0.01), social recognition: (r =0.367, p<0.05), WTM acquisition: (r =-0.513, p<0.01), WTM reversal: (r =-0.434, p<0.01) and FST climbing: (r =0.439, p<0.01). This suggests that decreased β-catenin levels in the NAc were associated with enhanced startle response, impaired social recognition, impaired performance in the WTM, and decreased climbing in the FST. A significant correlation also was found between mPFC β-catenin levels and climbing in the FST: (r =0.419, p<0.01), suggesting that lower β-catenin levels in the mPFC were associated with decreased climbing (i.e., decreased coping behavior) (*, p<0.05; **, p<0.01).

Figure S4. The effects of URB597 and AM251 on exploration time during the social preference and the social recognition tests

A two-way ANOVA (shock×drug; 2×2) on total exploration time revealed significant main effects of shock [preference: F(5,42)= 47.4., p<0.001; recognition: F(5,42)= 5.641, p<0.05] and drug [preference: F(5,42)= 6.134., p<0.01; recognition: F(5,42)= 6.248, p<0.01], with no significant drug×shock interactions. Post hoc analysis revealed that during the social preference test, the Shock/Veh and the Shock/am+URB groups showed decreased exploration time compared with their NoShock groups (NoShock/Veh and No Shock/am+URB groups, respectively: both p<0.001). Also, the NoShock/am group demonstrated decreased exploration time compared with the NoShock/Veh group (p<0.01) and the NoShock/am+URB group (p<0.001). For social recognition, the NoShock/Veh group demonstrated increased exploration time compared with the NoShock/am group (p<0.001), the NoShock/am+URB group (p<0.01), and the Shock/am group (p<0.01). Hence, the exploration time of the shocked rats co-administrated with URB597 and AM251 was similar to shocked rats treated with vehicle, suggesting that the effects of URB597 on exploration time were blocked by AM251 treatment (**, p<0.01; ***, p<0.001).

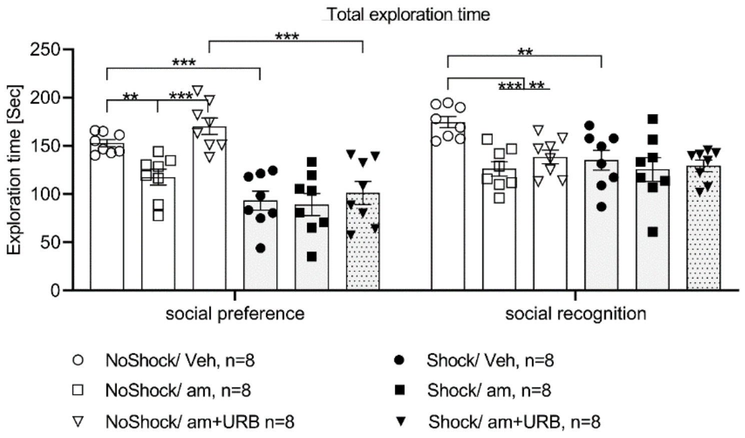

Figure S5. The effects of NAc β-catenin overexpression on exploration time during the social preference and the social recognition tests

A two-way ANOVA (shock×virus; 2×2) on exploration time revealed significant main effects of shock [preference: $F(3,30)= 6.567$, $p<0.05$] and drug [preference: $F(3,30)= 37.558$, $p<0.001$; recognition: $F(3,30)= 14.218$, $p<0.01$], with a significant drug×shock interaction [recognition: $F(3,30)= 16.610$, $p<0.001$]. Post hoc analysis revealed that during the social preference test, the GFP groups (NoShock/GFP and Shock/GFP) demonstrated decreased exploration time compared with the OE groups (NoShock/OE and Shock/OE, respectively; both $p<0.01$). Also, the Shock/GFP group demonstrated decreased exploration compared with the NoShock/GFP group ($p<0.01$). During the social recognition test, the Shock/GFP group showed decreased exploration compared with both the NoShock/GFP group ($p<0.01$) and the Shock/OE group ($p<0.001$). This suggests that NAc β-catenin overexpression prevented the shock- and reminders-induced decrease in exploration (**, $p<0.01$; ***, $p<0.001$).

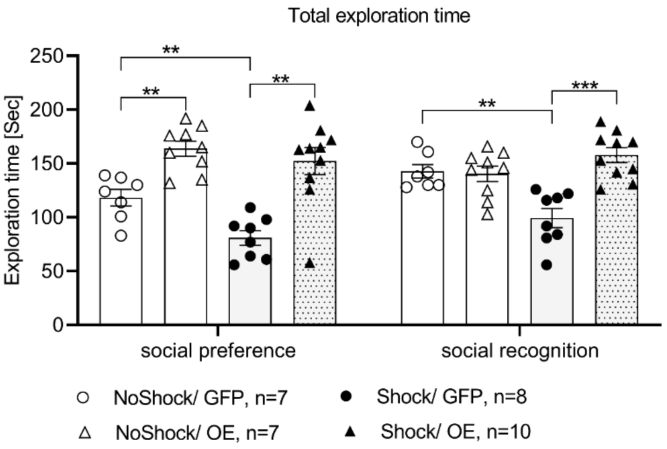

Figure S6. The effects of NAc β-catenin downregulation on exploration time during the social preference and the social recognition tests

A three-way ANOVA (shock×virus×drug; 2×2×2) on exploration time revealed a significant main effect of shock [preference: $F(7,76)= 46.203$, $p<0.001$; recognition: $F(7,76)= 21.836$, $p<0.001$] and a significant shock×virus interaction [preference: $F(7,76)= 6.734$, $p<0.05$]. Post hoc analysis revealed that during the social preference test, the Shock/GFP+Veh, Shock/DR+Veh and Shock/DR+URB groups demonstrated decreased exploration compared to their respective non-shocked controls (NoShock/GFP+Veh, $p<0.01$; NoShock/DR+URB, $p<0.001$; and NoShock/DR+Veh, $p<0.05$). Also, the Shock/DR+URB group showed decreased exploration compared with the Shock/GFP+URB group ($p<0.05$). During the social recognition test, the Shock/DR+Veh group showed decreased exploration compared to NoShock/DR+Veh group ($p<0.01$) (**, $p<0.01$; ***, $p<0.001$).

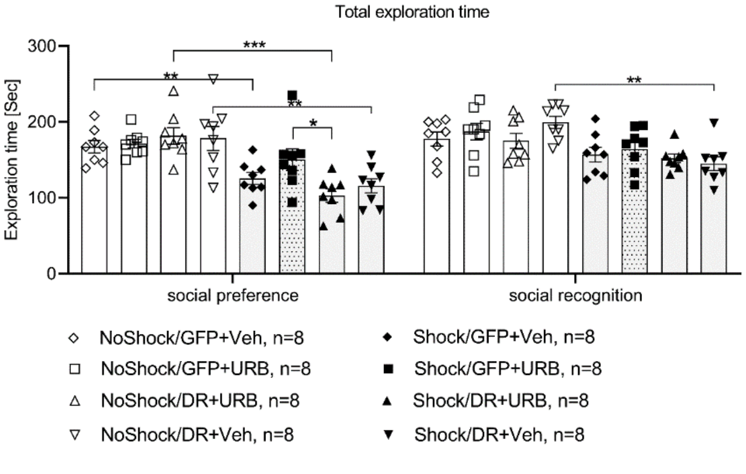

Supplement: Supplementary file 1 [file biomedicines-10-01789-s001.zip › biomedicines-1760907-supplementary.pdf]
